# Supplementary material for: Detection of Ultra-Rare ESR1 Mutations in Primary Breast Cancer Using LNA-Clamp ddPCR
Source: Cancers (Basel). 2023 May 6;15(9):2632. doi: 10.3390/cancers15092632 (PMC10177270; doi:10.3390/cancers15092632)
Supplement: Supplementary file 1 [file cancers-15-02632-s001.zip › cancers-2342865-supplementary.pdf]

Supplementary Materials: Detection of Ultra-Rare *ESR1* Mutations in Primary Breast Cancer Using LNA-clamp ddPCR

Yoko Hashimoto, Nanae Masunaga, Naofumi Kagara, Kaori Abe, Tetsuhiro Yoshinami, Masami Tsukabe, Yoshiaki Sota, Tomohiro Miyake, Tomonori Tanei, Masafumi Shimoda and Kenzo Shimazu

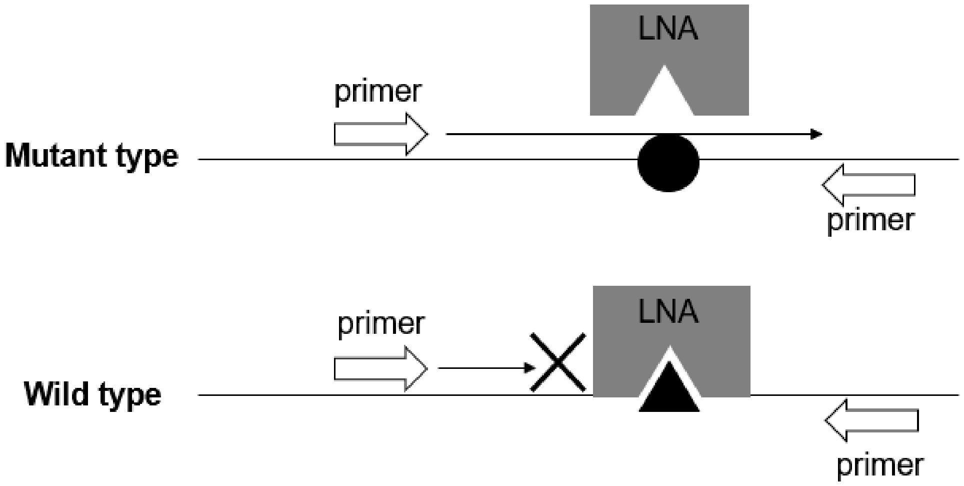

Figure S1. Description of LNA-clamp ddPCR. Schematic diagram of LNA-clamp ddPCR is shown. Arrows indicate foward and reverse primers, gray squares indicate LNA, and black circles indicate mutant sequences.

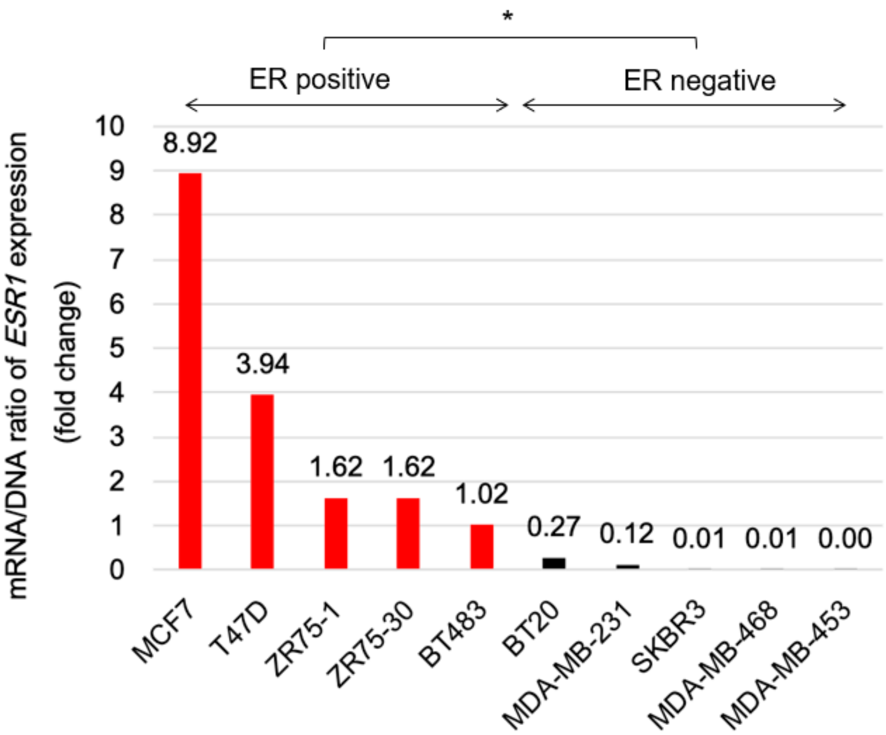

Figure S2. Ratio of the mRNA expression of *ESR1* to the DNA expression in breast cancer cell lines. The ratios of the mRNA expression of *ESR1* to the DNA expression of *ESR1* in 10 breast cancer cell lines are shown. *p < 0.05 by Wilcoxon signed-rank test. ER, estrogen receptor.

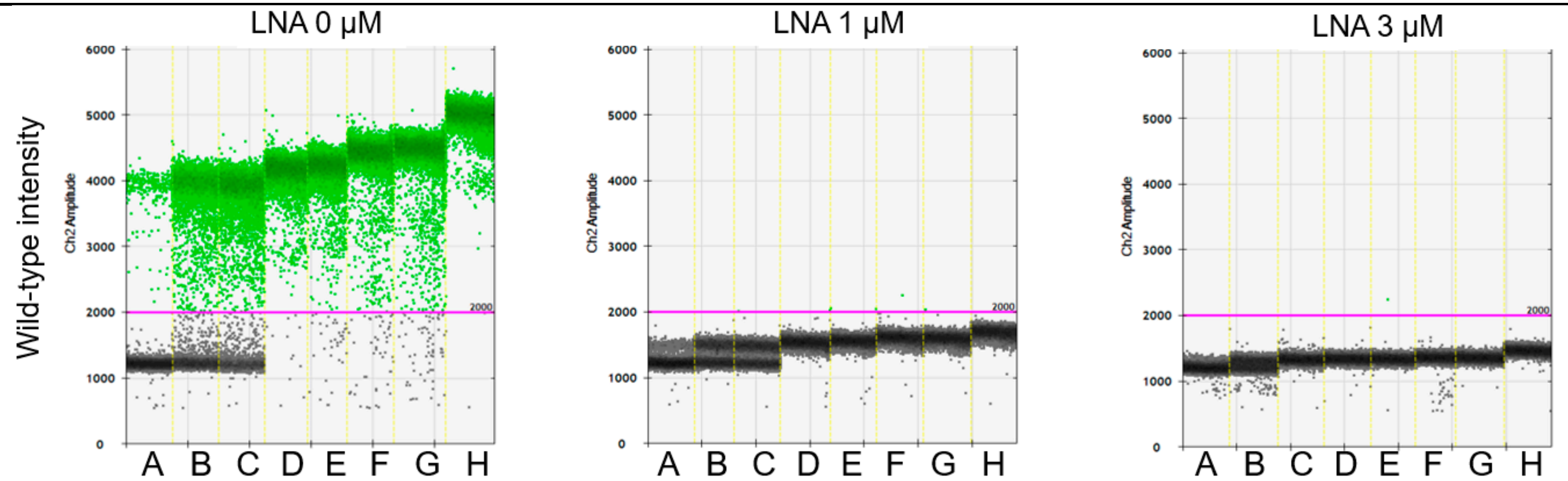

		A	B	C	D	E	F	G	H
Input copy number		6,000	30,000	60,000	300,000	600,000	3,000,000	6,000,000	60,000,000
LNA conc.	0 μM	484	15,900	32,660	166,000	138,000	135,000	130,200	210,000
	1 μM	0	0	1.4	2.6	1.4	2.8	1.4	0
	3 μM	0	0	0	0	1.4	0	0	0

(a)

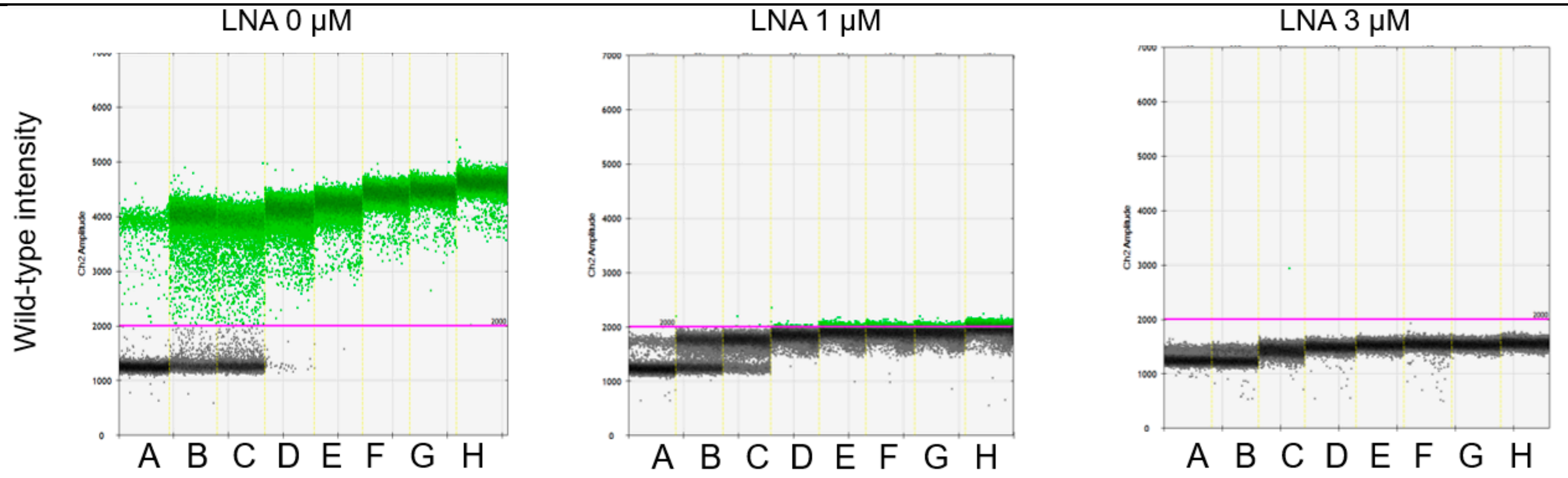

		A	B	C	D	E	F	G	H
Input copy number		6,000	30,000	60,000	300,000	600,000	3,000,000	6,000,000	60,000,000
LNA conc.	0 μM	802	30,700	18,300	145,600	230,000	>20,000,000	>20,000,000	>20,000,000
	1 μM	0	1.4	10.8	138	1038	1022	996	7280
	3 μM	0	0	1.4	0	0	0	0	0

(b)

Figure S3. Clamping effect of LNA-oligo. The clamping effect of LNA-oligo was measured using wild-type oligo (6,000–60,000,000 copies). (a) Effect of Y537S LNA-oligo. (b) Effect of D538G LNA-oligo.

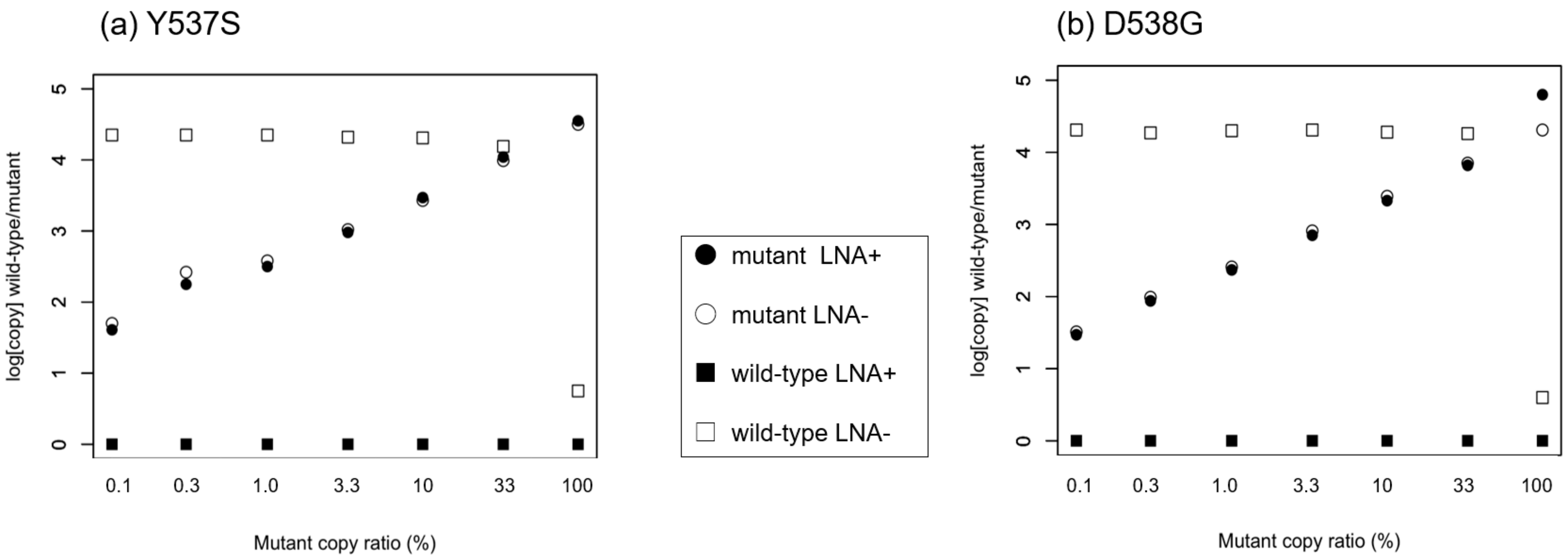

Figure S4. Sensitivity test of the PCR clamping method, Y537S (a) and D538G (b) Sensitivity test with serially diluted Y537S mutant DNA in wild-type DNA (100%, 33%, 10%, 3.3%, 1.0%, 0.3%, 0.1%, 0%). (a) Effect of Y537S LNA-oligo. (b) Effect of D538G LNA-oligo.

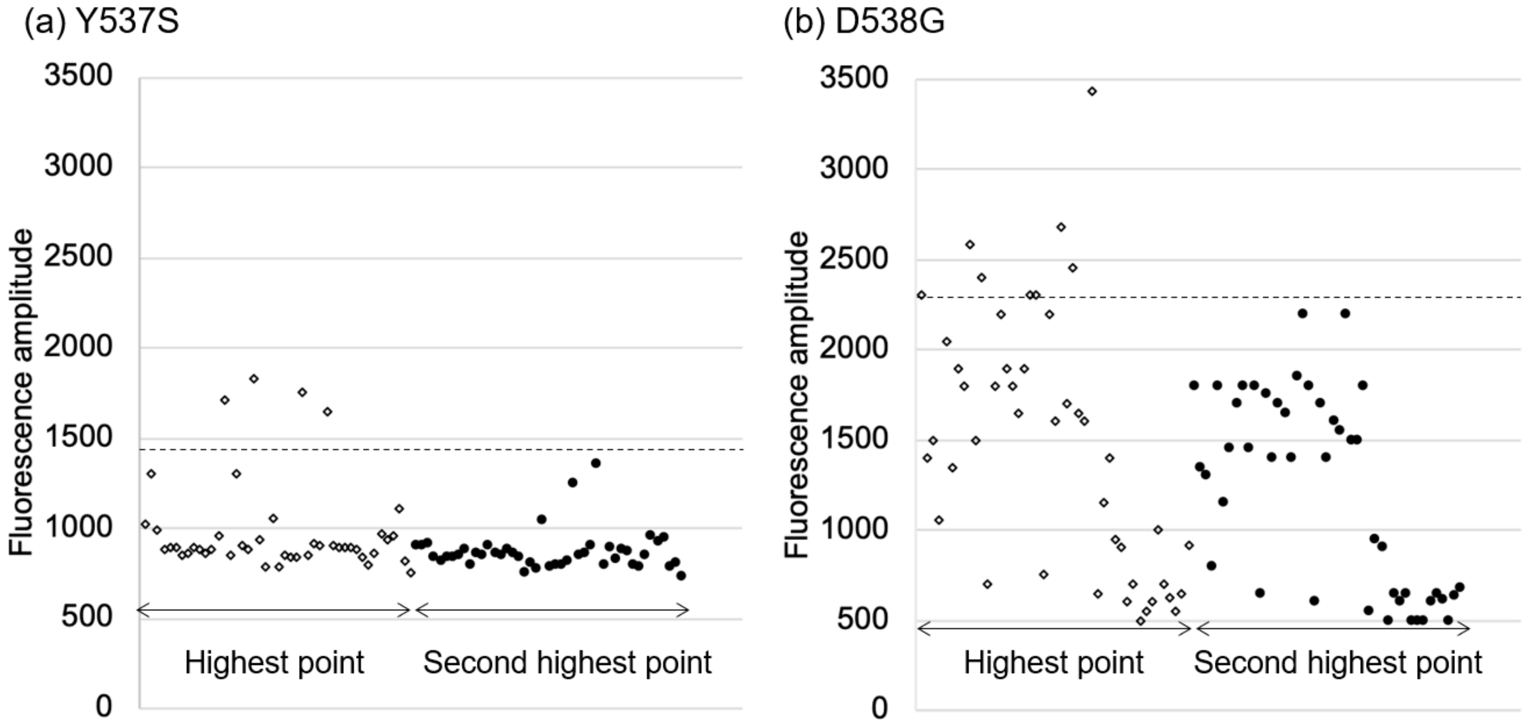

Figure S5. Backgrounds of LNA-clamp PCR, Y537S (a) and D538G (b) Backgrounds of LNA-clamp PCR were analyzed by cDNA from 26 normal breast fresh-frozen tissues and 19 WBC from healthy controls. The fluorescence amplitude of the highest point (white diamonds) and second highest point (black circles) of each wells are plotted. (a) Background of Y537S. (b) Background of D538G. Each dotted lines indicate the respective cutoff determined from the backgrounds.

Table S1. Primers, probes, mutant-oligos, and LNA-oligos for ddPCR.

**AA change**	**Y537S**	**D538G**
SNV	c.1610A>C	c,.1613A>G
Forward Primer	CAGCATGAAGTGCAAGAACGT	CAGCATGAAGTGCAAGAACGT
Reverse Primer	TGGGCGTCCAGCATCTC	TGGGCGTCCAGCATCTC
WT Probe	CCCCTCTATGACCTGC	CCCCTCTATGACCTGC
Mutant Probe	CCCTCTCTGACCTGC	CCCTCTATGGCCTGC
Mutant-oligo	CAGCATGAAGTGCAAGAACGTGGTGCCCCTCTCTGACCTGCTGCTGGAGATGCTGGACGCCCA	CAGCATGAAGTGCAAGAACGTGGTGCCCCTCTATGGCCTGCTGCTGGA-GATGCTGGACGCCCA
LNA-oligo	CCCTCT+A+TGACCTGC	CCCTCTATG+A+CCTGC

WT, wild type, oligonucleotides are given in 5'-3'order. + symbol denotes the LNA base.

Table S2. AI treatment for adjuvant setting.

		ESR1 **mutation positive (n=27)**	***ESR1*** **mutation negative (n=185)**
AI treatment for adjuvant setting	Yes	12	105
	No	15	80

AI, aromatase inhibitor.

Table S3. Treatment status of 13 recurrent cases.

		ESR1 **mutation positive (n=3)**	***ESR1*** **mutation negative (n=10)**
AI treatment	No	2	2
	Adjuvant	0	6
	Recurrence	1	5
DFI of recurrent breast cancer (month)	Median (range)	35 (22-60)	47 (7-90)

AI, aromatase inhibitor; DFI, disease free interval.

Table S4. Duration of AI for recurrence setting.

		ESR1 **mutation positive (n=1)**	***ESR1*** **mutation negative (n=5)**	***p***
Duration of AI for recurrence setting (month)	Median (range)	4	12 (4-36)	0.37*

AI, aromatase inhibitor, * Mann–Whitney U test.
